# Consensus‐based Guideline on tinea capitis

**DOI:** 10.1111/ddg.70395x

**Published:** 2026-07-14

**Authors:** Peter Mayser, Dietrich Abeck, Philipp P. Bosshard, Jochen Brasch, Georg Daeschlein, Isaak Effendy, Yvonne Gräser, Peter Höger, Julia Huynh, Maria Kinberger, Annette Kolb‐Mäurer, Katharina Antonia Langen, Bartosz Malisiewicz, Alexander Nast, Hagen Ott, Christin Pelzer, Dieter Reinel, Martin Schaller, Silke Uhrlaß, Pietro Nenoff

**Affiliations:** ^1^ Biebertal Germany; ^2^ Dermatology Office am Tibarg Hamburg Germany; ^3^ Department of Dermatology University Hospital Zurich University of Zurich Zurich Switzerland; ^4^ Department of Dermatology Venereology and Allergology Mycological Laboratory Schleswig‐Holstein University Hospital Campus Kiel Kiel Germany; ^5^ Medical University Brandenburg Medical University of Applied Sciences Anhalt Köthen Germany; ^6^ MVZ – Dermatology Franziskus Hospital Bielefeld Bielefeld Germany; ^7^ Charité – Universitätsmedizin Berlin, Corporate member of Freie Universität Berlin and Humboldt‐Universität zu Berlin Institute of Microbiology Infectious Diseases and Immunology, National Reference Laboratory for Dermatophytes Berlin Germany; ^8^ Catholic Children's Hospital Wilhelmstift Hamburg Hamburg Germany; ^9^ Charité – Universitätsmedizin Berlin, Corporate member of Freie Universität Berlin and Humboldt‐Universität zu Berlin, Department of Dermatology Venereology and Allergology Berlin Germany; ^10^ Charité – Universitätsmedizin Berlin, Corporate member of Freie Universität Berlin and Humboldt‐Universität zu Berlin, Department of Dermatology Venereology and Allergology, Division of Evidence Based Medicine (dEBM) Berlin Germany; ^11^ Department of Dermatology University of Würzburg Würzburg Germany; ^12^ Department of Dermatology, Venereology and Allergology Schleswig‐Holstein University Hospital, Campus Kiel Kiel Germany; ^13^ Main Medic All Health MVZ GmbH, Frankfurt am Main Frankfurt am Main Germany; ^14^ Section Pediatric Dermatology, MUC‐iSPZ Hauner, LMU Center for Children with Medical and Developmental Complexity Munich Germany; ^15^ Department of Dermatology, Venereology and Allergology, Cantonal Hospital St. Gallen HOCH Health Ostschweiz, St. Gallen Switzerland; ^16^ Dermatology Office Neuhaus Germany; ^17^ Department of Dermatology, University Hospital Tübingen Eberhard Karls University Tübingen Tübingen Germany; ^18^ labopart – Medical Laboratories Dr. Gerber, Prof. Dr. Bühling, Prof. Dr. Nenoff, T. Löwe, E. von Rein Mölbis Germany

**Keywords:** Tinea capitis, Mycological diagnostics, Antifungal therapy

## Abstract

The consensus‐based guideline developed under the auspices of the German Dermatological Society (DDG) is intended to provide physicians with a structured framework for clinical decision‐making in the management of patients with tinea capitis and to contribute to improved diagnostic accuracy, treatment adherence, and prevention.

Tinea capitis is a dermatophyte‐induced infection of the scalp that predominantly affects children and represents a common dermatological condition worldwide. In recent years, a marked shift in the epidemiology of causative dermatophytes has been observed in Europe, driven by migration, tourism, and socioeconomic factors, with an increasing prevalence of anthropophilic species, particularly *Trichophyton tonsurans* and *Trichophyton violaceum*.

From a therapeutic perspective, a combination of systemic and topical antifungal treatment is recommended, as topical monotherapy is generally insufficient. In pediatric patients, the choice of systemic antifungal therapy is primarily guided by the identified pathogen, with *Trichophyton* infections preferably treated with terbinafine (*off‐label*) and *Microsporum/Nannizzia* infections with itraconazole (*off‐label*) or griseofulvin. In adult patients, terbinafine is recommended as first‐line therapy irrespective of the causative species.

In addition, the guideline addresses measures to interrupt chains of infection, including the treatment of asymptomatic carriers, implementation of hygiene measures, and management of outbreak situations in community settings.

## INTRODUCTION

The present publication is a short version of the guideline. Several chapters are not included in this short version. Parts of the text of the included chapters are considerably shortened. The complete long version of the guideline is available on the AWMF website: https://register.awmf.org/de/leitlinien/detail/013‐033.

This consensus‐based guideline is an update of the German guideline published in 2019.^1^ The results of a PubMed search (accessed August 2024) were incorporated taking especially the current international guidelines into account, in particular the guidelines of the European Society for Pediatric Dermatology 2010^2^ and the British Association of Dermatologists 2014.^3^

### Definition

Tinea capitis (TC) is a dermatophyte infection of the scalp that predominantly affects children.^2^, ^3^

### Pathogens and transmission

*Microsporum (M.) canis* is the most common zoophilic pathogen worldwide, while *Trichophyton *(*T.) violaceum* and *T. tonsurans* are the predominant anthropophilic pathogens of TC.^4^, ^5^, ^6^ The frequency of the latter has increased globally in the last 20 years. This increase is driven, in particular, by socioeconomic factors as well as tourism and migration. Such a shift is also reflected in current epidemiological data from Europe, albeit with marked regional differences.^4^, ^5^, ^6^, ^7^, ^8^, ^9^, ^10^, ^11^, ^12^, ^13^, ^14^, ^15^, ^16^, ^17^, ^18^, ^19^, ^20^, ^21^, ^22^, ^23^, ^24^, ^25^, ^26^, ^27^, ^28^, ^29^, ^30^ Notably, there is a constant increase of anthropophilic species (*M. audouinii, T. tonsurans, T. violaceum, T. soudanense*) in Western and Northern Europe,^7^, ^8^, ^9^, ^10^, ^11^, ^12^, ^13^ as well as Switzerland^16^, ^17^ and Germany.^31^ The high infectivity of these pathogens and the possibility of an asymptomatic pathogen status may cause additional infections and small outbreaks.^4^, ^5^, ^6^

Apart from the other zoophilic pathogens relevant for TC, *T. mentagrophytes, T. quinckeanum*,^32^, ^33^, ^34^, ^35^ and *T. verrucosum*, *T. benhamiae* has also gained in importance.^36^ In kerion celsi, *M*. *canis* and *T*. *tonsurans* are the most common species, although other dermatophytes, such as *T. mentagrophytes* and *T. verrucosum*, may also be causative pathogens. ^37^, ^38^, ^39^ In Germany, *T. tonsurans* may currently not only be found in migrants but also in adolescent martial artists, especially in wrestling.^40^, ^41^ Currently, an increased incidence is observed in boys, male adolescents, and young men approximately 2–3 weeks after a shave or a haircut in so‐called barbershops. In some cases, contaminated barbershop equipment, such as razors and combs, insufficient hygienic measures, and microtraumas have been identified.^42^, ^43^, ^44^, ^45^ At present, *T*. *tonsurans* must be considered as “*emerging pathogen*” especially in TC. The infection rates have markedly increased since 2020.^46^

### Pattern of infection and clinical presentation

Based on clinical considerations and the type of infection identified by microscopy, dermatophytoses of terminal hairs (trichomycoses) are classified into tinea in the strict sense, microsporosis in the strict sense, and favus (tinea favosa).^47^, ^48^ TC is probably the most common dermatophytosis in childhood and occurs predominantly between the 3rd and 7th year of age; infants and neonates are rarely affected.^49^, ^50^, ^51^, ^52^ With puberty, TC becomes a rare event. In this context, fungistatic activity of the sebum triglycerides changed during puberty is discussed as important factor.^53^

Trichomycoses arise from spreading of the pathogens from the stratum corneum in hair follicle and shaft to the zone of keratinization (Adamson's fringe). Outward growth of the hair results in dissemination of spores. Pathogen and immune response determine the clinical type that may vary from mainly non‐inflammatory lesions with mild scaling to deeply infiltrating infections with abscess formation (kerion). Favus (tinea favosa) is characterized by shield‐like crusts (scutula) that may coalesce to larger masses. However, the clinical presentation does not allow for reliable identification of the causative pathogen. Clues may be obtained from the (microscopically detectable) form of the spores and their position relative to the hair.

At the hair, ectothrix and endothrix infection are distinguished. In the former, the fungus adheres predominantly to the surface of the hair shaft in the form of arthrospores, whereas in the latter, the pathogen penetrates into the hair shaft without destroying the cuticle. Typical pathogens for ectothrix infections are *M. canis* and *M. audouinii* (small spores, “microsporosis”) as well as *T. mentagrophytes* and *T. verrucosum* (large spores). Most anthropophilic pathogens (in particular *T. tonsurans, T. violaceum, T. soudanense*) result in endothrix infections of the hair. With intact cuticle, the hair is filled with spores and visible as a small black dot in the opening of the follicle (“*black‐dot ringworm*”) due to the trichomalacia. Given the position of the spores within the hair shaft, endothrix infections caused by anthropophilic pathogens are often of low immunogenicity. Frequently, the scalp presents only with pityriasiform scaling with absent or minor erythema. This will often result in an asymptomatic carrier status (in risk populations up to 15 %; particularly in adults) that may be relevant for disseminating the infection to other individuals.^54^ If dermatophytes penetrate along hair follicles into the depth, follicular pustules and, in some cases, massive purulent discharge potentially associated with general symptoms, such as fever and headache as well as swelling of lymph nodes, may occur. While this is often misinterpreted as bacterial abscess, secondary bacterial infections should not be overlooked. Disc‐like manifestations with massive purulent discharge (like “honey from a comb”) are called kerion (Greek for honeycomb) celsi (Aulus Cornelius Celsus, Roman encyclopedist and medical writer). Triggers are usually zoophilic dermatophytes, such as *T. verrucosum, T. mentagrophytes, T. benhamiae*, or the geophilic *Nannizzia gypsea* (formerly *M. gypseum*).^55^ In recent years, however, inflammatory manifestations of endothrix infections, caused in particular by *T. tonsurans* or *T. violaceum*, have also been observed in urban regions.^56^

### Microsporosis

Microsporosis in the strict sense is a clinically almost unreactive deep folliculitis without abscess formation caused by *M. audouinii* (anthropophilic) or *M. canis* (zoophilic),^57^ less often by other *Microsporum/Nannizzia* species (spp.), such as *M. ferrugineum* (anthropophilic)^58^ or *Nannizzia gypsea* (geophilic). Prepubescent children are almost exclusively affected. An inflammatory reaction is usually completely absent. In the circular, sharply delimited lesions that appear as if dusted with flour, the dull hairs are broken just above the level of the scalp. The size of the lesions varies; they may occur as single or multiple lesions and may coalesce in the latter case. Microscopic examination of hair stubs usually reveals a large number of small spores forming a sheath around the hair shaft (diameter 2–3 µm), which is why the disease is called microsporosis. Yellow‐green fluorescence under Wood's light confirms the diagnosis of *Microsporum/Nannizzia* infection.

### Favus

In Europe, favus is very rarely encountered. Nevertheless, individual cases have been described, especially from Northern Africa and Tunisia. Similarly, individual cases of favus caused by *Trichophyton schoenleinii* continue to occur in Germany.^59^ Favus is characterized by shield‐like scaly crusts, so‐called scutula, containing mycelial masses and developing in and around the hair follicle, enclosing one or several hair shafts in the center, and healing with scarring alopecia (pseudopelade condition). An “offensive” odor resembling mouse urine, completes the symptoms. 95 % of those affected have clinical symptoms, especially scutula, bleaching of hairs, atrophy, and scarring. The peak of incidence is in children aged from 6–10 years (predominantly in boys), but the disease does not disappear when they reach puberty.^60^ While it may persist for life, the average disease duration is five years (10 days to 59 years). In 7 % of the tinea favosa cases, the remaining skin and the nails are also affected (onychomycosis favosa).

Because the pathogen grows within the hair shaft (endothrix favus), favus is less infectious than other dermatophytoses. On the other hand, it is also located in a niche that is difficult to access by infection defense mechanisms and environmental factors. Accordingly, the pathogen may survive for up to 54 months in epilated hairs.

In general, TC does not cause permanent loss of hair. Irreversible destruction of the hair follicle with scarring alopecia (pseudopelade condition) may occur due to a destructive inflammatory reaction, particularly in TC profunda or due to infection of *T. schoenleinii* (favus).

If TC is suspected based on clinical findings, a full‐body examination for additional manifestations of dermatophytosis, including onychomycosis, should be performed in addition to thorough history assessment.^61^ Moreover, recurrent manifestations of dermatophytosis or onychomycosis in children may be caused by autoinoculation. Conversely, TC can lead to onychomycosis of the fingernails and, less commonly, the toenails. This tinea unguium is a direct consequence of scratching the affected scalp. While manifestation of TC in adults is less common, the incidence is increasing.^3^, ^14^, ^19^ The pathology occurs more often in women than men; hormonal disorders, immunosuppression, autoinoculation, and transmission by affected children have been suggested as causative factors. Unusual clinical appearance and course,^62^ “*side‐cut*” TC after shaving in barbershops caused by *T. tonsurans*,^42^, ^43^, ^63^, ^64^ and pathogens rather atypical for the disease in children, such as *T. rubrum*. ^65^ should be considered in the diagnostic work‐up.

In TC, dermatophyte‐induced dermatophytids can exhibit significant clinical variability as a manifestation of a delayed‐type reaction to the pathogen.^66^, ^67^ Criteria for a dermatophytid include: 1. Verified, often inflammatory dermatophytosis on a localization free of tinea (here, TC); 2. No evidence of fungal elements or DNA in dermatophytid‐related lesions; 3. Often, initial aggravation on therapy with systemic antifungal medications (release of large amounts of antigens from the pathogen). This must be differentiated from a drug reaction against the systemically administered antifungal medication. 4. Resolution after therapy of the dermatophytosis.^68^, ^69^ Generally, dermatophytids may appear on the trunk, extremities and face.^70^ Generalized reactions are also possible. The following reactions are described in TC: symmetric disseminated small papular or papulo‐vesicular eczematous lichenoid reactions with follicular accentuation usually on the trunk (lichen trichophyticus), erythema nodosum and erysipelas‐like dermatitis, psoriasiform lesions, erythema multiforme, as well as erythema annulare centrifugum‐like and urticarial lesions.^66^, ^67^, ^71^, ^72^, ^73^, ^74^

### Differential diagnosis

The differential diagnosis of TC is very wide and includes all pathologies with patchy hair loss, scaling, or inflammation of the scalp.^1^, ^3^, ^47^ Psoriasis capitis, seborrheic and atopic eczema of the scalp, secondary stage of syphilis (alopecia areolaris specifica), as well as exsiccation eczema and pityriasis amiantacea (“tinea amiantacea”) are sometimes difficult to differentiate from forms of TC with low‐grade inflammation. Alopecia areata usually presents without scaling, but sometimes with inflammation. Exclamation mark hairs need to be differentiated from the broken hairs in TC. Lupus erythematosus, lichen planopilaris, and trichotillomania have to be considered, particularly if scarring is observed. Inflammatory forms of TC may be misinterpreted as bacterial folliculitis, abscesses (furuncle, carbuncle), folliculitis decalvans, or eosinophilic pustular folliculitis of infancy. A less common differential diagnosis is cutaneous T‐cell lymphoma in childhood.

## DIAGNOSTIC WORK‐UP

The diagnosis of TC is based on clinical and laboratory examinations.^48^, ^75^, ^76^ The former consist predominantly of medical history (course, additional cases in the neighborhood, animal contacts, stays abroad), inspection (type of infection, additional clinical manifestations of dermatophytosis, such as tinea corporis, onychomycosis) and, if necessary, examination by UV light (∼365 nm, so‐called Wood's light) and dermatoscopy. Laboratory tests consist of direct microscopical (wet mount) and, if necessary, histological examination (in particular with PAS staining) as well as pathogen identification in culture and by PCR.^77^ Pathogen identification is important for choosing the antifungal medication and detecting infection chains. Examination with Wood's light may be particularly helpful in case of outbreaks and for detecting affected animals. If yellow‐green fluorescence is observed, the diagnosis of *Microsporum* infection (for example, by *M. canis*) is confirmed. However, given that the sensitivity for *Microsporum* infection of the scalp is rather low, the examination is not suitable for a diagnosis of exclusion.

### Collection of material

Optimal sampling is a prerequisite for valid test results.^47^ Sterile equipment should be used for this purpose. When using selective growth media with cycloheximide and antibiotics, prior disinfection of the collection site is not required. Suspected lesions can be identified either clinically or, if necessary, by use of a Wood's lamp or trichoscopy. In case of deep inflammatory lesions, any pruritic crusts should be removed with forceps and hair stubs should be pulled out from the lesion edge. These can be easily pulled out (with sterile forceps or a needle holder) due to the highly inflammatory lesions. In lesions with low inflammatory activity, sufficient amounts of scales and hairs can be collected from the scalp either with the blunt side of a scalpel or by brush or swab methods. With the brush method, hair and scalp are combed several times with a (sterile) scalp brush which is then pressed gently directly onto the agar, so that material adhering to the bristles is transferred to the culture medium. The number of positive impressions allows for an approximate quantification.^78^

### Microscopy and pathogen determination

For direct microscopy (“wet mount”), the material on the slide is overlaid with 10 % to 20 % potassium hydroxide (KOH) or, for better contrasting, with a suitable dye (for example, 0.025 % Congo red in 5 % sodium dodecyl sulphate (SDS) solution, Parker ink, or Chicago sky blue). The test material is covered by a coverslip and the specimen is transferred for 10–30 minutes into a moist chamber. The specimen is screened under the microscope at approximately 10x (overview) and 40x objective lens magnification and 10x eyepiece magnification.^47^ Detection of (in dermatophytes colorless) hyphae and spores in the wet mount indicates fungal infection, but cannot provide reliable information about the pathogenic species. With good preparation and a fairly short incubation in KOH or dye in order to avoid too severe damage of the hair structure, ectothrix and endothrix fungal infection of the hairs can be distinguished. The intrinsic color and form of fungal elements may also be apparent, which may be helpful for differentiation from some molds. While the sensitivity of wet mounts is not very high, it can be increased by addition of optical brighteners (Calcofluor white®) and evaluation by fluorescence microscopy.^47^, ^79^

The following growth media are suitable for cultivating fungi from hair roots and scales:^79^ Sabouraud glucose agar with 2 or 4 % glucose, Kimmig agar, and agar containing antibiotics and cycloheximide (for example, Dermasel® agar from Thermo Fisher Scientific, Schwerte, Germany) or dermatophyte agar (for example, from Sifin, Berlin, Germany). The additives are useful for suppressing growth of bacteria and molds that would otherwise result in fast overgrowth of the slower growing dermatophyte. For inoculation, scales and hairs should be transferred onto the agar and hair stubs should be “planted” with the root into the growth medium. The fungal culture is stored at 26–28 °C for three to four weeks, or up to six weeks if infections of *T. verrucosum, T. violaceum*, or *T. soudanense* are suspected, and controlled macroscopically for fungal growth once per week.^80^ While an incubation temperature of 37 °C accelerates the growth of *T. verrucosum*, it is too high for other dermatophytes.^77^

Species are identified based on macroscopic evaluation of thallus morphology and color, microscopic determination of macroconidia and microconidia, and other typical growth forms, such as chlamydospores or spiral hyphae.

### Molecular methods for pathogen detection in TC

Molecular methods, such as conventional PCR with subsequent species identification by sequencing or use of specific probes (ELISA format, Microarray, Blot), real‐time PCR, or MALDI‐TOF MS (matrix‐assisted laser desorption/ionization time‐of‐flight mass spectrometry), are highly sensitive and specific methods suitable for both differentiation of a pathogen from primary culture and (PCR methods) direct pathogen identification from clinical material. With the latter, a result may be obtained within 24 hours. With MALDI‐TOF MS, common dermatophytes can be identified rapidly and reliably from the fungal culture, provided the required database is available, of course.^81^, ^82^, ^83^, ^84^, ^85^, ^86^ However, it should be noted that especially in the case of *T*. *mentagrophytes* complex, differentiation of the included species and genotypes is not yet possible.^87^ Apart from in‐house procedures, commercially available test systems (kits) are increasingly used for this purpose.^88^, ^89^, ^90^ Currently, at least 5 commercial test systems are available, including a multiplex PCR microarray for simultaneous detection of at least 23 dermatophytes and additional relevant non‐dermatophyte fungi (3 yeasts and molds each) by hybridization after amplification of fungal DNA in just one reaction step. Molecular techniques are, therefore, significantly faster (24–48 hours versus 2–6 weeks for culture‐based methods) and more sensitive than wet mount and culture.^73^ However, it is usually not possible to recognize or differentiate rare or newly described species with these tests. False negative results are also possible, for example, due to inhomogeneous distribution of fungal elements in the test material (fungal elements usually occur in nests). Accordingly, the source material – dandruff and hair roots, possibly swabs – should always be collected in sufficient quantity and prepared accordingly. Equally, the PCR result should always be compared critically with the clinical presentation, given that it is always possible, due to the high sensitivity of the method, that DNA contamination of collection equipment, collection room, or lab area may result in contamination of the sample (false positive results). With diligent work especially in the molecular biological laboratory, however, the risk of contamination can almost be ruled out.

The extent to which PCR may be suitable for assessing the therapeutic course of TC, in addition to its use in rapid pathogen identification, is currently unknown and requires further analysis. Our own experience shows that after combined topical and systemic antifungal therapy of TC caused by *T*. *tonsurans*, growth is still observed in culture up to two months after treatment discontinuation while PCR may still be positive after even three months. This is also reflected in the conclusion of a recently published study from Switzerland where PCR was considered inappropriate for therapy control in TC due to its high sensitivity.^91^ There was a significantly more plausible correlation of treatment success with fungal culture. Further studies on the usefulness of PCR for follow‐up and treatment success are urgently needed.

### Histology

In pre‐treated patients with repeatedly inexplicable negative mycological test results, histological examination of biopsies in combination with a fungal stain (PAS, Grocott‐Gomori's methenamine silver stain, Calcofluor white) and possibly PCR may be useful.^92^, ^93^ Histological examination is particularly beneficial in TC profunda or in favus,^59^ given that the obtained scales may contain very few fungal elements. Moreover, PCR‐based diagnosis may also allow for identification of the dermatophyte species in formalin‐fixed biopsies.^93^

### Dermatoscopy/trichoscopy

According to the results of recent studies, trichoscopy – as a tool in TC diagnostics – allows for a simple, rapid, non‐invasive diagnosis and is particularly useful under conditions where mycological analysis is not available. Based on characteristic findings, it may also provide clues as to the type of infection (ectothrix, endothrix) and thus guide the choice of therapeutic agent, but it does not provide definite identification of the pathogen.^75^, ^94^, ^95^, ^96^

### Resistance testing

Since an increased development of resistance in dermatophytes has not yet been demonstrated, routine resistance testing of dermatophytes isolated from TC is not currently warranted.^97^, ^98^

## THERAPY

Basically, therapy of TC pursues four goals that need to be coordinated in a treatment plan with all parties involved:^1^, ^2^, ^3^, ^99^

1. Clinical and mycological cure with reliable pathogen elimination as fast as possible (negative wet mount and culture);
2. Rapid elimination of symptoms;
3. Avoidance of permanent hair loss;
4. Prevention of further transmission or interruption of infection chains.

TC should always be treated systemically and, in addition, topically; this is usually managed in an outpatient setting. Hospitalization due to TC is only rarely required (severe cases) and almost exclusively in kerion celsi. Surgical interventions in case of abscess formation on the scalp with differential diagnosis “mycosis” without completed mycological diagnostic workup are contraindicated.^100^, ^101^

### Treatment start

Targeted therapy requires an exact diagnosis with identification of fungi in wet mount and culture or by PCR‐based methods. Exceptions apply for initial treatment in severe cases if there is a justified suspicion (even without waiting for “rapid diagnosis” by PCR and if ambiguous microscopic results are obtained) and if there is a suspected infection with resistant strains with high risk of transmission based on a case‐by‐case assessment.^3^ For the interruption of infection chains, the quickest possible diagnosis should be used primarily (wet mount + PCR). Culture parallel to PCR would be optimal.

Identification of the pathogen is a decisive factor for selection, type and duration of administration, and dose of the antifungal medication. For systemic therapies, the age of the patient, in children also the body weight, and potential interactions with other medications should be included in the treatment plan. If a zoophilic species is detected, animals that may be responsible for transmission must undergo veterinary examination and testing for fungi even if there are no signs of infection, and must be treated with antifungal medication if positive results are obtained. Further contact of the animals to the patient and his environment must be avoided.

### Topical therapy

While topical monotherapy is usually not sufficient for the treatment of TC,^1^, ^2^, ^3^ topical preparations should always supplement the therapy. They support killing of pathogens even in localizations not reached by systemic antifungal agents, resulting in reduction of spores and infectivity, shorten the duration of systemic therapy, and are first‐line medications for treating asymptomatic carriers with low burden of spores.^78^, ^102^, ^103^, ^104^ Suitable agents for topical treatment of the affected scalp include certain azoles, amorolfine, and ciclopirox olamine, the latter preferably as solution. Terbinafine‐containing cream is an alternative. Creams and solutions are applied twice daily; alternatively, two different agents may be applied alternately in the morning and evening; for example, ciclopirox olamine and clotrimazole.^105^ In addition, shampoos and/or solutions with antifungal activity containing selenium (di)sulfide, ketoconazole, clotrimazole, ciclopirox olamine, or piroctone olamine should be used. Shampoos should be applied for at least five minutes twice weekly over a period of 2–4 weeks. Restricting the application duration of the antifungal agent is not useful. Its application duration should only be limited after clinical and mycological response. Especially in infancy and early childhood, special instructions for the use of topical antifungal agents according to the summary of product characteristics must be taken into account.

While these medications are covered by insurance in Germany for the treatment of TC in children up to age 12, the costs for treating asymptomatically infected individuals and their close contacts must be borne by the patients themselves. Accordingly, the cost factor may play a role in patient compliance. This is important given that co‐treatment of asymptomatic carriers is essential for containment of the infection, especially in infections caused by anthropophilic species, such as *T. tonsurans* or *M. audouinii*.^1^, ^2^, ^3^ These situations often involve outbreaks, in which case the public health department may also be considered for cost coverage.

### Systemic therapy

In principle, all systemic antifungal agents are significantly more effective in case of endothrix infections (for example, *Trichophyton spp*.) compared to ectothrix infections (for example, *M. canis*). In general, antifungal therapy should always be guided by the identified pathogen (Table 1).^104^, ^106^, ^107^, ^108^, ^109^, ^110^, ^111^, ^112^, ^113^, ^114^, ^115^, ^116^, ^117^, ^118^

**TABLE 1:** Selection of oral antifungal medication depending on the isolated pathogen for the treatment of TC in children (before puberty).

*Trichophyton mentagrophytes, benhamiae, tonsurans, violaceum, soudanense* and other *Trichophyton spp*.	Terbinafine
*Microsporum canis, M. audouinii, M. ferrugineum*	Itraconazole or griseofulvin (no longer available)^*^
*Nannizzia gypsea*	Itraconazole or griseofulvin (no longer available)^*^

* Griseofulvin is no longer marketed in Germany, Austria, and Switzerland, but continues to be recommended internationally, particularly for Microsporum/Nannizzia infections, and is the only antifungal approved in Germany for children with TC

TC caused by *Trichophyton* species should be treated primarily with terbinafine, and TC caused by *Microsporum/Nannizzia spp*. with itraconazole (Table 1).^1^, ^2^, ^3^, ^105^ In the latter genera of dermatophytes, griseofulvin is equivalent to itraconazole for TC in children. However, given that all griseofulvin preparations are no longer marketed in Germany and many other European countries (Belgium, Greece, Portugal, Turkey, Austria, Switzerland) since 2018 and have to be purchased inconveniently via the International Pharmacy (individual imports according to § 73 German Medicinal Products Act [*Arzneimittelgesetz*, AMG]), prescriptions with this medication have declined substantially.^105^

Treatment initiation before definite identification of the pathogen should take the local epidemiology and the most likely pathogenic species into account.

For reasons of both pharmaceutical legislation and pharmacokinetics, a distinction must be made between treatment of adults and children.^1^ For oral treatment of adults, the antifungal agents terbinafine, itraconazole, and fluconazole are available and approved for the treatment of “dermatomycoses (for example, tinea)” (Table 2). At present, griseofulvin is the only medication approved for children.

**TABLE 2:** Oral treatment of TC in adults.

Drug	Dosage per day	Treatment duration^1^
Itraconazole	100–200 mg^2^ once daily immediately after a main meal	4 weeks
SUBA (Super‐Bioavailability) Itraconazole	50 mg twice daily (*off‐label*)	4 weeks
Fluconazole	50 mg once daily, alternatively 150 mg once weekly	4–7 weeks 4–8 weeks
Terbinafine	250 mg once daily	4–6 weeks

^1^ The stated treatment durations are rough indications,^105^ The actual duration depends on the clinical presentation and the results of mycological testing, which should be performed every 4 weeks starting from week 4 of treatment. The goal is mycological cure. Additional topical treatment (see below) is essential even in adults. Cure is generally expected faster in adults than in children.

^2^ According to the manufacturer's information, a dose of 100–200 mg daily is recommended.

In Germany, fluconazole as well as terbinafine and azoles are generally not approved for treatment of dermatophyte infections in children (*off‐label‐use*). Therefore, based on the AMG, administration of fluconazole and the two other oral antifungal agents, terbinafine and itraconazole, in children is only possible as an individual therapeutic trial (Table 3).

**TABLE 3:** International recommendations for the treatment of TC in children.^1–3,104,106,107^

**Drug**	**Dosage**	**Treatment duration for Trichophyton spp**.^5^	**Treatment duration for Microsporum/Nannizzia spp**.^5^
Itraconazole^1^, ^2^	5 mg/kg body weight once daily with a main meal; or for < 20 kg BW 50 mg/day for > 20 kg BW, 100 mg/day for < 10 kg body weight: strictly weight‐adapted dosing	4 weeks	6 weeks
Fluconazole^1^, ^3^	3–6 mg/kg BW 1x daily	3–4 weeks 4–8 weeks	6–8 weeks
Terbinafine^1^	< 20 kg body weight: 62.5 mg/day 20–40 kg body weight: 125 mg/day > 40 kg body weight: 250 mg/day	4 weeks	8–12 weeks Role in the treatment of these pathogens is controversial
Griseofulvin (ultramicronized)^4^	10–15 mg/kg body weight once daily (for M. canis higher dose of 20–25 mg/kg), in one or two divided doses daily with a main meal	6–8 weeks	8–12 weeks


^1^ Not approved for use in children; in Germany only applicable as an individual therapeutic trial. Data on terbinafine use in children under 10 kg body weight (under 1–2 years) are limited to isolated case reports.^121^

^2^ Maximum daily dose regardless of body weight: 100 mg/day. An oral suspension formulation has not been available since February 1, 2024. Alternatively, after consultation with the respective manufacturer, a weight‐adjusted amount of a 100 mg capsule can be compounded into a suspension in a pharmacy. For smaller children, a liquid formulation of another active substance is preferable.

^3^ Fluconazole is generally not approved in Germany for the treatment of dermatophyte infections in children. However, with parental consent, an individual therapeutic trial may be justified.

^4^ Generally approved for children. Infants and toddlers up to 2 years only in justified exceptional cases. Not to be used in neonates. Griseofulvin is no longer marketed in Germany.

*Average minimum duration; goal = negative fungal detection (culture/PCR).

^5^ The stated treatment durations are guideline values. The actual duration depends on the clinical presentation and the results of mycological testing, which should be performed every 2–4 weeks starting from week 4 of treatment. Often, significantly longer treatment periods of 3 to 4 months are required – not only for kerion celsi but also, for example, in particularly dry hyperkeratotic forms of TC.^105^

If griseofulvin is not used, it is mandatory to inform the legal guardian(s) in detail about the lack of approval for the prescribed medication for the age group, as well as the advantages of the *off‐label* therapy compared to the only medication approved in Germany, griseofulvin, in addition to other potential antifungal agents or even non‐treatment, with consideration given to possible risks and side effects, potential unknown side effects, and the most severe possible courses. Adequate documentation is also mandatory.^119^ In Austria and Switzerland, terbinafine is approved for the treatment of children from the 2nd year of age. In children, newer antifungal agents are, therefore, currently always administered in the context of an individual therapeutic trial according to the AMG. Numerous studies have shown, however, that the newer antifungal agents terbinafine and itraconazole are superior to griseofulvin with respect to response rates, therapeutic safety, and cost effectiveness.^104^, ^106^, ^107^, ^108^, ^109^, ^110^, ^111^, ^112^, ^113^, ^114^, ^115^, ^116^, ^117^, ^118^, ^120^

### Reasons for the inadequate response in children

In the study by Ghannoum et al.,^97^ terbinafine showed strong antifungal efficacy in vitro to 301 isolates of dermatophytes from patients with TC worldwide, including *Microsporum canis* (n = 94) and *M. audouinii* (n = 19). The findings do not adequately reflect clinical reality insofar as systemically administered terbinafine, in particular, is not the first‐line medication in children with *Microsporum* infections due to the low healing rates. Based on the data of Ghannoum et al., this is not caused by reduced in vitro sensitivity or resistance of the pathogens. The inadequate response in children is rather caused by the special anatomical and physiological characteristics of children before puberty (dry skin, absent lipid and fatty acid production of sebaceous glands) and the mechanism of action of both azoles and allylamines on the fungal cell.^122^

### Treatment strategy in the event of TC and follow‐up^1^, ^2^, ^3^, ^123^

1. If a positive result is obtained in wet mount and/or culture/PCR and/or Wood's light testing, initiation of combined systemic and local antifungal therapy, at first standardized for four weeks. Selection of substance based on history/epidemiology, result of mycological diagnosis (specimen, culture, and PCR).
2. Four weeks after therapy initiation, repeat complete mycological diagnostic workup (with PCR, if available). Until culture findings are available (maximum duration of incubation four weeks), interruption of systemic treatment with continued local therapy if there is a marked improvement of the clinical findings and always if there is apparent regrowth of hairs (if necessary, verification by trichoscopy). Otherwise, both forms of treatment should be continued.
3. If culture (and possibly PCR) is still positive, continue topical and systemic therapy for another 2–4 weeks (until next mycological testing).
4. Repetition of this procedure (items 2–3) until culture finding is negative. Goal and endpoint of treatment is the mycological cure, which is therefore guided by the individual courses of the patients.

Monitoring of laboratory parameters: Traditionally, liver function tests (ASAT, ALAT, GGT) have been performed under systemic antifungal therapy. The benefit of this procedure is doubtful, however, given that severe hepatotoxic changes, in particular, occur within a very short time window and are difficult to detect during a routine check after 4–6 weeks.^124^, ^125^, ^126^ On the other hand, it is known for all substance groups that impairment of liver function may occur (albeit with different frequency/severity), in rare cases with serious to lethal outcome. For these reasons, targeted education, precise knowledge of the patient's individual status, and meticulous overall clinical monitoring – not just of hair status – are of particular importance during the course of antifungal treatment, which often lasts for months.

Therefore, the following approach is recommended (Table 4).

**TABLE 4:** Laboratory monitoring during systemic antifungal therapy (allylamines, azoles, griseofulvin).

**Patients with known impairment of liver function, hepatotoxic co‐medication, or other comorbid diseases affecting liver function**	AST (ASAT), ALT (ALAT), and GGT before starting therapy, after 2–4 weeks, and thereafter individually depending on the course.
Patients without risk factors	Currently, there is no consensus regarding the necessity of laboratory monitoring. The decision should be made and documented on an individual basis together with the patient, depending on dosage, duration of therapy, and possibly additional factors.
Patients or their legal guardians must be informed and this must be documented as part of the consent process about possible side effects, in particular symptoms of potential liver damage. This is of high legal relevance for the treating physician, especially when using non‐approved drugs in off‐label therapy. If indicative gastrointestinal symptoms occur, therapy should initially be interrupted, and the patient should undergo thorough evaluation, including laboratory testing. Treatment interruption or discontinuation may also be required in cases of more severe immunological intolerance reactions. A prerequisite for safe treatment is regular clinical monitoring (follow‐up visits) and the possibility for the informed patient to make contact in case of suspected adverse effects.	

### Additional measures

#### Exclusion from school and kindergarten

After reviewing the data and based on our own experience, children receiving appropriate systemic and adjuvant topical therapy may return to school or kindergarten. In case of highly contagious anthropophilic pathogens (*T. violaceum, T. soudanense*, potentially *T. tonsurans, M. audouinii*) a waiting period of one week may be considered. Anthropophilic fungi are more infectious and contagious, and they spread easily from a child with a relatively dry, scaly TC to other children. Irrespective of this rule, children with purulent, abscess forming TC and kerion celsi should of course stay at home until wound healing is completed.

An asymptomatic carrier status may contribute to persistence of infection chains. For anthropophilic pathogens, examination and topical treatment of all family members and close contact persons is recommended even in the absence of clinical findings, preferably with antifungal shampoos. For zoophilic pathogens, screening and, if necessary, treatment of animals in cooperation with a veterinarian is recommended.

#### Shaving of hair/wearing of headdress

For all forms of TC in children, the time required until infectivity is lost under treatment is also dependent on the length of the affected hairs. Cutting back or shaving the hairs may significantly reduce the treatment duration with systemic antifungal medication. However, given the topical combination therapy with highly effective fungicidal antimycotics, this procedure is no longer required, as is the recommendation to wear a head cap.

### Screening within the family

Especially diseases caused by anthropophilic pathogens, such as *T. tonsurans, T. violaceum, T. soudanense*, and *M. audouinii*, are highly contagious. All family members and contact persons should be interviewed and examined for symptoms.^127^ More than 50 % of family members including adults may be affected, often as asymptomatic carriers.^78^ Non‐treatment of symptomatic or asymptomatic family members may result in high recurrence rates. Accordingly, mycological testing and, if necessary, treatment of all family members, including asymptomatic members, and close contact persons is recommended.

After reviewing the data and based on our own experience, it is concluded that administration of systemic antifungal medication in contact persons is only recommended if there are actual signs of manifest TC. In asymptomatic contact persons, topical antifungal therapy preferably with antifungal shampoos should be performed. Shampoos should be applied for at least five minutes twice weekly over a period of four weeks. The underlying reasons should be explained in detail to ensure compliance.^127^ Repeated mycological controls (every four weeks) are required in symptomatic patients to ensure treatment success.

### Procedure in the event of an outbreak/reporting obligation

Dermatophyte infections are generally exempted from reporting in accordance with the German Infection Protection Act (IfSG § 34). There is, however, a reporting obligation in the framework of regulations for community settings, if two or more similar diseases with a likely or suspected epidemic, temporal, or spatial connection occur, and if this indicates a serious threat to the community.

### Treatment of contaminated objects and inventory

Vital spores of anthropophilic species have been isolated from hairbrushes and combs.^128^, ^129^ Accordingly, items of personal hygiene, such as combs, hair brushes, razors, towels, washcloths, bed linen, scarfs, headdresses, stuffed animals, and toys must always be disinfected or discarded, or must be replaced by items for single use, if fungal infection is suspected.^1^, ^130^ In addition, home furnishings, such as curtains and furniture/upholstered furniture, have to be disinfected appropriately, if contamination by fungal elements is likely, for example, in kindergartens or in case of pets that may be triggers for TC infections with zoophilic pathogens. If zoophilic pathogens are identified, pets should be examined and treated by a veterinarian. Pets need not be sick, but may be asymptomatic carriers for the respective pathogens.^36^, ^131^

### Use of glucocorticoids

According to some authors, development of maximally inflammatory TC (kerion) depends also on the formation of a strong delayed‐type immune response to the pathogen, indicated by the frequent formation of dermatophytids.^68^, ^69^ Therefore, use of oral or intralesional glucocorticoids has been recommended in the literature to accelerate the healing process and minimize the risk of permanent alopecia.^132^, ^133^ An increased immune reaction or pruritus caused by highly potent antifungal monotherapy and rapid disruption of pathogens in case of inflammatory forms of TC can be addressed initially with a topical combination therapy of glucocorticoids and antifungal agents for a limited period of approximately seven days.^132^, ^133^

### Therapy failure

Reasons for therapy failure may include:

1. Non‐compliance – especially in case of long treatment duration;^1^, ^2^, ^3^
2. Underdosage (especially too low dosage for too short treatment duration or reduced efficacy of oral antifungal agents due to co‐medication);^134^, ^135^
3. Relative resistance of the pathogen (insufficient drug concentration at the target site);^122^
4. Resistance of the pathogen (especially, *T*. *mentagrophytes* ITS genotype VIII / *T. indotineae* due to mutations in the squalene epoxidase gene);^105^, ^136^, ^137^, ^138^, ^139^, ^140^
5. Reinfection;
6. Underlying diseases of the affected individual resulting in impaired immune response.

If there is no clinical improvement, it is important to ensure that the chosen antifungal therapy is adequate for the detected pathogen (Table 1). Otherwise, the following options are available:

1. Increase dosage or therapy duration of the original antifungal agent;
2. Switching of antifungal agent (Table 1), especially if an unexpected pathogen has been detected).

## CONFLICT OF INTEREST STATEMENT

See long version of the guideline: https://register.awmf.org/de/leitlinien/detail/013‐033.
